# Production, partial optimization and characterization of keratinase enzyme by *Arthrobacter* sp. NFH5 isolated from soil samples

**DOI:** 10.1186/s13568-017-0462-6

**Published:** 2017-09-21

**Authors:** Nirmal Chandra Barman, Fatema Tuj Zohora, Keshob Chandra Das, Md. Golam Mowla, Nilufa Akhter Banu, Md. Salimullah, Abu Hashem

**Affiliations:** 1 Department of Biotechnology and Genetic Engineering, Faculty of Applied Science and Technology, Islamic University, Kushtia, 7003 Bangladesh; 2 Microbial Biotechnology Division, National Institute of Biotechnology (NIB), Ganakbari, Savar, Dhaka, 1349 Bangladesh; 3 Molecular Biotechnology Division, National Institute of Biotechnology, Savar, Dhaka, 1349 Bangladesh; 4 Department of Biology and Chemistry, North South University, Bashundhara, Dhaka, 1229 Bangladesh

**Keywords:** *Arthrobacter* sp., Keratinase, Enzyme activity, Optimization, Characterization

## Abstract

The study was conducted to select the best promising keratinolytic bacterial strain. A good keratinase positive bacterium isolated from the soil samples of Hazaribagh tannery industrial zone, Dhaka was identified as *Arthrobacter* genus depending on the conventional techniques and confirmed as *Arthrobacter* sp. by sequencing 16S rRNA gene. The medium components and culture conditions were optimized to enhance keratinase production through shake flask culture. Keratin and feather powder (10 g/l or 1%) were good substrates for the highest keratinase production along with yeast extract (0.2 g/l or 0.02%) as an organic nitrogen source and potassium nitrate (1 g or 0.1%) as an inorganic nitrogen source. Maximum yield of keratinase was found after 24 h of incubation at 37 °C with an initial pH of 7.0 and inoculums volume 5% under 150 rpm when keratin, yeast extract and potassium nitrate were used as nutrient sources. Keratinase production was more than 5.0-fold increased when all optimized parameters were applied simultaneously. The optimum reaction temperature and pH were determined to be 40 °C and 8.0 respectively for crude keratinase activity. Therefore, *Arthrobacter* sp. NFH5 might be used for large scale production of keratinase for industrial purposes in less time.

**Electronic supplementary material:**

The online version of this article (doi:10.1186/s13568-017-0462-6) contains supplementary material, which is available to authorized users.

## Introduction

A huge amount of feather and leather wastes are generated every year as a by-product from the poultry and tannery industry due to continuous meat consumption worldwide. Globally over 5 million tons of feathers are produced from poultry-processing plants as a waste product (Poole et al. 2009) every year. Feathers contain a very high content of about 90% keratinous protein, (Onifade et al. 1998; Martinez-Hernandez and Velasco-Santos 2012). These keratins are biologically insoluble, fibrous, recalcitrant and biochemically rigid molecules which are resistant to degradation by most common proteolytic enzymes (McGovern 2000; Riffel et al. 2007; Zaghloal et al. 2011). Native keratin is also highly inert and exhibits usually un-degradable characteristics and lead to persist long term in the environment.

Tannery industries are continuously using large amount of chemicals for dehairing in leather processing. These dehairing chemicals contain a high amount of pollutants such as soda-lime, salts, chromate, sulphide, solvent flashy, emulsified fatty matter, waste lime liquor. Beside these biological wastes from leather itself causing environmental pollution (Pepper and Wyatt 1989). The chemicals used in leather processing are also corrosive and health hazardous. Some tanneries have been forced to close down due to their pollution effect in environment (Davighi 1988). Very recently, government of the People’s Republic of Bangladesh has taken an initiative to move the tannery industry from Dhaka city; and build up the tannery industry outside Dhaka city and modernize the industry.

Hence, microbial and biotechnological approach could be a promising and better alternative to keratin and keratinous wastes recycling by enzymatic action using specific enzyme of microorganism namely keratinase (Mehta et al. 2014). The potential applications of such microbial keratinases have been reported (Gupta and Ramnani 2006; Brandelli 2008; Chojnacka et al. 2011). The hydrolysate of keratinous proteins i.e. amino acids could be used as animal feed and many other applications.

Keratinases (3.4.21-/24/99) are the enzymes that can hydrolyze a number of keratinous materials. Bacterial keratinases have potentiality because of their activity on degradation of specific insoluble keratin substrates and generally on a variety of protein materials (Lin et al. 1996). This enzymes work as novel biocatalysts that have many applications in several sectors including in leather, textile and detergents industries, enhancing drug delivery system and medical application, in cosmetics, prion degradations, as pesticides, production of biodegradable films or bioprocessing of used X-ray film, in glues and foils and agro-industrial waste degradation (Saber et al. 2010; Mazotto et al. 2011a, b; Mohanapriya et al. 2014; Mohorcic et al. 2007; Gupta et al. 2002; Zaghloul et al. 2004).

A group of microorganisms such as bacteria, actinomycetes, saprophytic fungi and dermatophytes are found in different ecological environments which are able to produce keratinase enzymes and degrade keratinous waste (Lucas et al. 2003; Kansoh et al. 2009; Rahayu et al. 2010; Tork et al. 2010). Among them *Arthrobacter* sp. exhibit a great keratinolytic activity. The intention of present study was to isolate, identify and characterize keratinase producing bacteria of natural sources and to optimize keratinase enzyme production condition. The enzyme was also partly characterized in the study.

## Materials and methods

### Isolation of bacteria from local soil sample

The soil samples were collected from Hazaribagh tannery industry, Dhaka and local poultry farms of Savar, Dhaka. Then bacteria were isolated by serial dilution and spread plate technique. The keratin agar (KA) plates were incubated at 37 °C for 5–6 days. The bacterial isolates were further sub-cultured to obtain pure culture. The modified KA medium contains (g/l): NaCl (0.5 g), KH_2_PO_4_ (0.7 g), K_2_HPO_4_ (1.4 g), MgSO_4_·7H_2_0 (0.1 g), Keratin (ceratin) (10 g) and agar (15 g) with pH 7.5 (Agrahari and Wadhwa 2010).

### Screening the keratinase positive bacteria

Keratinase positive bacteria were cultured on skim milk agar and feather meal agar plates. The composition of skim milk agar medium was (g/l): casein 5.0 g, glucose 1.0 g, skim milk powder 3.0 g, yeast extract 2.5 g and agar 15.0 g as solidifying agent. The pH was adjusted to 7.5. Bacterial isolates were inoculated onto plates and incubated at 37 °C for 48 h. The clear zone forming isolates were selected as keratinase producer. 10% trichloroacetic acid (TCA) was flooded on the milk agar plate to observe the zone clearly (Saran et al. 2007).

Selected keratinase positive bacteria were further confirmed by using feather meal powder in the medium instead of keratin. The isolates those produce clear zones on both media were considered as keratinase producers.

### Identification of keratinase positive bacteria

Morphological and a range of biochemical tests were performed in order to identify the isolate (Accession Number KY593174; Strain Number CGMCC 1.16131). The isolate was identified based on morphological, biochemical characteristics as described in the Bergey’s manual of systematic bacteriology and by 16S rRNA gene sequencing.

Primers set used to amplify 16S rRNA sequence were **27f (**5′-AGA GTT TGA TCC TGG CTG AG-3′) and **1492r** (5′-GGC TAC CTT GTT ACG ACT T-3′) as forward and reverse primer-respectively in a PCR thermal cycler (ICycler 170-8740, USA). The thermal cycling program was initial denaturation at 95 °C for 5 min, followed by 30 cycles, denaturation at 95 °C for 30 s, annealing at 56 °C for 30 s, extension at 72 °C for 90 s and the final extension at 72 °C for 10 min. The amplified DNA was visualized by gel electrophoresis (Additional file 1: Figure S1). The 16S rDNA sequence was analyzed and compared with other deposited sequences in the Genbank via the online programme BLAST (http://www.ncbi.nlm.nih.gov/). Neighbor-joining phylogenetic tree was constructed based on the 16S rRNA sequences using MEGA6 (Tamura et al. 2013).

### Bacterial inoculums preparation

50 ml of nutrient broth (13 g/l at pH 7.4 ± 0.2) was prepared and sterilized in an autoclave (CL-40 M, Japan) at 15 lbs/in.^2^ pressure, 121 °C for 20 min. After cooling the media at room temperature, freshly grown single colony was transferred aseptically to it and incubated at 37 °C overnight at 150 rpm in a rotary shaking incubator (Stuart SI 500, UK).

### Enzyme production in shake flask cultures

The keratinase enzyme production was carried out in the basal medium by using shaking flask. The composition of the media was (g/l): feather meal powder (10 g), NH_4_Cl (1 g), NaCl (1 g), K_2_HPO_4_ (0.6 g), KH_2_PO_4_ (0.8 g), MgCl_2_·6H_2_O (0.48 g) and yeast extract (0.2 g) with pH 7.5 (Rajesh et al. 2014). 2.5 ml of the bacterial inoculums was added in 50 ml of medium and cultured on a rotary shaking incubator (Stuart SI 500, UK) at 150 rpm and 37 °C for 72 h. After incubation, fermented broth was centrifuged at 5000 rpm for 20 min at 4 °C. The cell free supernatant was collected and used for the assay of keratinase activity.

### Enzyme assay

The enzyme activity was determined by keratin digestion method using 1% keratin in 0.05 M Tris–HCl buffer (pH 8.0) as substrate according to Cai et al. (2008). The reaction mixtures contain 1.75 ml substrate solution and 0.25 ml crude enzyme solution. Then mixture was incubated at 40 °C in water bath for 10 min and reaction was terminated by adding 2.0 ml 0.4-M trichloroacetic acid (TCA). The control also was made by incubating the enzyme solution with 2.0 ml TCA without addition of keratin. The mixture was then centrifuged at 3825 rpm for 30 min and absorbance was measured at 280 nm by spectrophotometer (Jenway 6305, USA) against the control. One unit (U/ml) of enzyme activity is defined an increase of absorbance of 0.01 at 280 nm (*A*280) (Gradišar et al. 2005) per minute under the assay condition calculated by the following equation.

1 $$ {\text{U}} = 4\times n \times A 2 80/\left( {0.0 1\times 10} \right) $$

where *n* is the dilution factor; 4 is the final reaction volume (ml); 10 is the incubation time (min).

### Optimization of cultural conditions for keratinase production

The production of keratinase by bacterial inoculums was studied by considering the media components and culture conditions. All the experiments were carried out in triplicate and the mean values were presented.

### Effects of substrates on keratinase production

Various substrates such as keratin, casein, peptone, skim milk powder and feather meal powder were used (10 g/l) as main nutrient sources separately for the production of keratinase. Fermentation was carried out with 5% inoculums at 37 °C for 72 h at 150 rpm. The pH and volume of the media were 7.5 and 50 ml respectively.

### Effects of organic and inorganic nitrogen sources on keratinase production

The keratinase production by the isolated bacterium strain was also optimized by supplementing different organic and inorganic nitrogen sources individually. The organic nitrogen sources such as tryptone, peptone, beef extract, gelatin and yeast extract were used as 0.02% concentration as well as NH_4_NO_3_, KNO_3_, NH_4_H_2_PO_4_, NH_4_SO_4_ and NH_4_Cl were used 0.1% concentration as inorganic nitrogen sources in the media.

### Effects of temperature and pH on keratinase production

To determine the suitable temperature for enzyme production, the culture media were incubated from 28 to 52 °C (28, 33, 37, 42, 47, 52 °C) temperature and for determination of optimum pH for keratinase production experiments were carried out from 5 to 10 (5, 6, 7, 7.5, 8, 9, 10) separately. The pH was adjusted by using 0.1 N HCl or 0.1 N NaOH. Fermentation was carried out for 72 h as stated earlier.

### Effects of incubation period and inoculum volume on keratinase production

The effect of incubation period on keratinase production was examined by carrying out fermentation up to 24, 48, 72 and 96 h separately. Different inoculums volumes (2, 3, 4, 5, 6, and 7%) in media were also used to determine the appropriate inoculum volumes require for maximum production of enzyme.

### Effects of agitation speed on keratinase production

The effect of agitation speed for keratinase production was studied at 120, 140, 150, 160 and 180 rpm independently. The assay was conducted after 72 h.

### Partial characterization of crude keratinase activity

The optimum pH for enzyme activity were determined by incubating the reaction mixture at various pH range from 5 to 10 at shaking water bath for 10 min at 40 °C using 0.05 M Tris–HCl buffer and the effect of temperature on enzyme activity were checked by incubating the reaction mixture at temperatures from 30 to 80 °C (30, 40, 50, 60, 70, 80 °C) by using same buffer for 10 min and assayed for enzyme activity.

### Keratinase production at optimized condition

After standardization of all production parameters, enzyme production was carried out at optimum condition and compared with initial production rate.

## Result

### Isolation and identification of bacteria

**Fig. 1:**
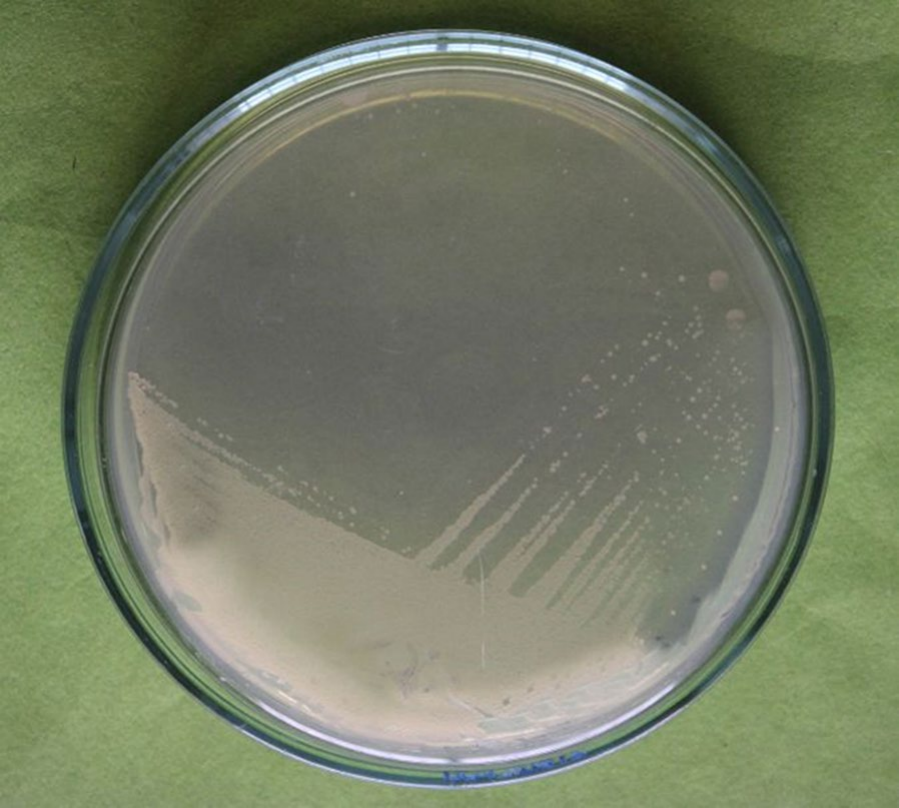
Photograph of the bacteria growth

**Fig. 2:**
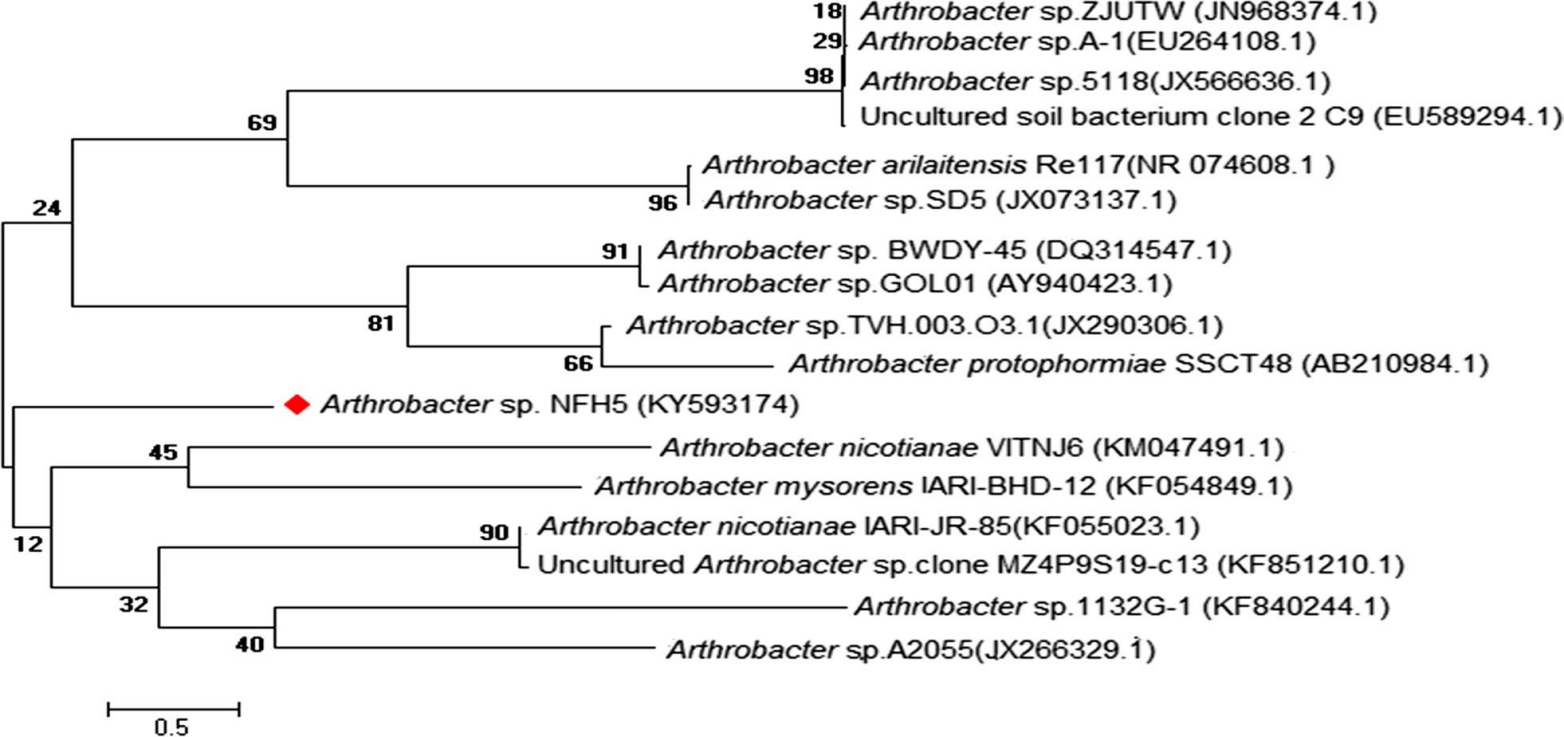
Phylogenetic tree based on 16S rRNA gene sequence and showing the relationship between *Arthrobacter* sp. NFH5 with other selected members of *Arthrobacter* sp. The phylogenetic tree was deduced by using the Neighbor-Joining method (Saitou and Nei 1987) and this finest tree is consisted with the total branch length of 19.36227215. The bootstrap test was conducted using 1000 replications and has been shown next to the branches (Felsenstein 1985). The same units such as length have been used throughout the phylogenetic tree. Maximum Composite Likelihood method and base substitution’s unit number was used to compute the evolutionary distances (Tamura et al. 2004). The study involved 17 nucleotide sequences. Codon positions comprised were 1st + 2nd + 3rd + noncoding. Entire locations enclosing gaps and missing data were removed. There were a total of 1438 positions in the final dataset

A total of 44 morphologically well-formed single colonies were selected from ten different soil samples on the basis of their morphological difference in nutrient agar plates. Among 44 tested bacterial isolates, 27 were found as keratinase producers based on their clear zones formation on skim milk agar and feather meal agar media. The isolates those formed the distinct clear zone on both media were considered as keratinase producing bacteria. Among all of them, best one keratinase producing bacterium NFH5 has been selected based on their enzyme activity. The bacterium was off-white, moderate round, entire margin and microscopically gram positive, non spore forming and cocci shaped (Fig. 1). The isolate was identified as *Arthrobacter* sp. based on morphological and biochemical characteristics and confirmed by 16S rDNA sequencing (Table 1). We denoted the isolate as *Arthrobacter* sp. NFH5 (strain number CGMCC 1.16131). The 16S rRNA gene sequence was compared to the Genbank of database using the BLAST. The 16S rRNA gene sequence of the isolate NFH5 showed high levels of sequence similarity with members of the genus *Arthrobacter* (Fig. 2; Additional file 1: Figure S2).

**Table 1:** Morphological and physiological characteristics of isolate *Arthrobacter* sp. NFH5

Morphological characteristics	
Shape	Cocci
Gram staining	Positive
Endospore	Non spore forming
Physiological characteristics	
Indole production	–
Methyl red	–
Voges–Proskauer	+
Citrate utilization	–
Oxidase	–
Catalase	+
Nitrate reduction	+
Starch hydrolysis	–
Gelatin hydrolysis	–
Growth on MacConkey agar	+
Glucose	–
Mannitol	–
Arabinose	–

### Optimization of growth conditions for maximum keratinase production

**Fig. 3:**
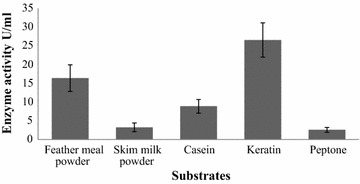
Effect of different substrates on keratinase production by *Arthrobacter* sp. NFH5. The fermentation was carried out at 37 °C with pH 7.5 at 150 rpm for 72 h. Data represent the means ± standard deviations for triplicate

The enzyme production was assayed by bacterium before optimizing the parameter of media components and culture condition. In our study, the production of enzyme by *Arthrobacter* sp. NFH5 was found 13.06 U/ml initially after 72 h fermentation carrying out. Maximum production was 26.57 U/ml in presence of 1.0% keratin in media and keratin-based substrates such as 1.0% feather meal powder gave yield of 16.40 U/ml which is second higher production (Fig. 3). The effects of different organic and inorganic nitrogen sources were also investigated.

**Fig. 4:**
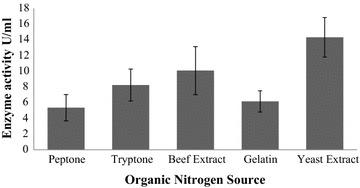
Effect of organic nitrogen source on keratinase production by *Arthrobacter* sp. NFH5. The fermentation was carried out at 37 °C with pH 7.5 at 150 rpm for 72 h. Data represent the means ± standard deviations for triplicate

**Fig. 5:**
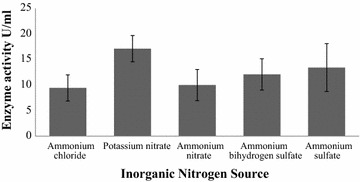
Effect of inorganic nitrogen source on keratinase production by *Arthrobacter* sp. NFH5. The fermentation was carried out at 37 °C with pH 7.5 at 150 rpm for 72 h. Data represent the means ± standard deviations for triplicate

Among the organic nitrogen supplemented media, maximum amount of keratinase production was found 14.32 U/ml in presence of 0.2 g/l (0.02%) yeast extract and minimum production was observed in peptone (5.36 U/ml) (Fig. 4). The highest keratinase production (17.13 U/ml) was found in 0.1% potassium nitrate supplemented media and lowest keratinase production (9.44 U/ml) was found in ammonium chloride supplemented media (Fig. 5).

Then the effects of different culture parameters were standardised. Higher amount of keratinase enzyme was found to produce by *Arthrobacter* sp. NFH5 strain after 24 h of incubation period at temperature 37 °C with pH 7.0 (Table 2). The maximum enzyme production was recorded 18.79 and 26.44 U/ml using 5% inoculums volume and 150 rpm agitation speed respectively.

**Table 2:** Effect of culture conditions for production of extracellular Keratinase from *Arthrobacter* sp. NFH5 in shake-flask cultivation

Culture condition	Specific activity (U/ml)	Relative activity (%)
Incubation period (h)		
24	25.8	100
48	19.56	75.81
72	15.02	58.22
96	6.92	26.82
Incubation temperature (°C)		
28	4.52	19.72
33	8.72	48.85
37	17.85	100
42	12.08	67.68
47	6.32	35.41
52	5.04	28.24
Initial pH		
5.0	5.08	20.56
6.0	10.03	40.62
7.0	24.69	100
7.50	18.37	74.40
8.0	12.84	58.0
9.0	9.48	38.39
10.0	5.92	23.98
Inoculums volume (%, ml)		
2.0	2.08	11.06
3.0	3.44	18.30
4.0	10.26	54.60
5.0	18.79	100
6.0	9.65	51.35
7.0	4.48	23.84
Agitation speed (rpm)		
120	8.72	32.98
140	12.08	45.69
150	26.44	100
160	12.72	48.11
180	6.16	23.30

Data represent the means of triplicate

The production of keratinase was achieved a significant amount when all optimized media components and culture conditions were applied together for fermentation. The yield of enzyme was fivefold increased (69.40 U/ml) of initial level after applying all optimized parameters at a time.

### Partially characterization of crude keratinase Activity

**Fig. 6:**
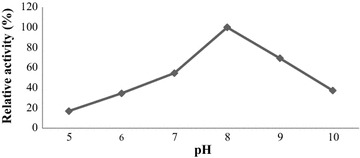
Effect of pH on keratinase activity. Enzymatic reaction was carried out at different pH ranging from (5 to 10) for 10 min at 40 °C in a shaking water bath and results are presented on graph. Data represent the means of triplicate

**Fig. 7:**
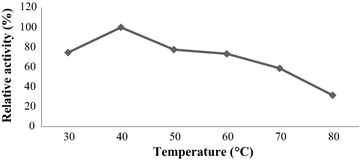
Effect of temperature on keratinase activity. Enzymatic reaction was carried out at temperatures from 30 to 80 °C for 10 min in shaking water bath and results are presented on graph. Data represent the means of triplicate

The activity of keratinase enzyme was determined by carrying out the reaction. The enzyme was more active between pH 7.0–9.0 where the optimum pH was determined at 8.0 (Fig. 6). The crude enzyme exhibited activity from neutral to alkaline conditions (pH 7.0–9.0) which is the range value of keratinase positive bacteria. Keratinase activity was also observed in a range temperature at 30–60 °C. Optimum temperature for keratinase activity was found at 40 °C. Increasing temperature up to 60 °C retains the relative activity over 70% as shown (Fig. 7).

## Discussion

In the present study, bacteria were isolated from collected soil samples and screened for keratinase producing capability on the basis of clear zone formation. The higher clear zone forming isolate on both media was considered as better keratinase producer. The bacterial isolate was gram positive and confirmed as *Arthrobacter* sp. (NFH5). In most cases, keratin degradation is executed by gram positive bacteria (Gupta and Ramnani 2006). It was also found in previous study that keratinases are produced by coccus *Arthrobacter* sp. (Pereira et al. 2014). *Arthrobacter creatinolyticus KP015744* has been reported as better keratinase producer (Kate and Pethe 2014). Thus *Arthrobacter* sp. appears to be a potential candidate for keratinase production.

Microbial keratinase is an inducible enzyme (Malviya et al. 1992). The isolated *Arthrobacter* sp. NFH5 produced maximum keratinase when keratinous protein elements were present in the media. Similar result was found for *Arthrobacter creatinolyticus KP015744* that gave highest keratinase production in presence 1% feather powder (Kate and Pethe 2014). Kainoor and Naik (2010) also achieved the maximum keratinase production in presence of 1% feather meal with *Bacillus* sp. JB99. The production of keratinase depends on the presence of keratin and its concentration. Enzyme production might be declined in the presence of higher concentration of feather meal indicating catabolic suppression (Saibabu et al. 2013).

Yeast extract as organic N_2_ source and potassium nitrate as inorganic N_2_ sources gave the maximum amount of enzyme production. It has been reported previously, maximum keratinase production was achieved in yeast extract supplemented media as organic nitrogen source (Sivakumar et al. 2013). In each case, lower amount of keratinase production was achieved from Peptone containing media. Temperature, pH and other culture parameters play vital role for enzyme production. Hence, optimum enzyme production by *Arthrobacter* sp. NFH5 was found at 37 °C. Kate and Pethe (2014) reported that maximum keratinase production was achieved by *Arthrobacter creatinolyticus* at 37 °C. The higher enzyme production was also found at 35 °C for 24 h by *Arthrobacter* sp. A08 (Pereira et al. 2014). Maximum temperature for keratinase production was recorded at 40 °C for some other keratinase positive bacteria such as *Bacillus subtilis* and *Bacillus pumilis* in previous result (Suh et al. 2001).

pH of the media affect the reaction environment, enzymatic process and transport of nutrients across the cell membrane of bacteria. Maximum production takes place when a suitable pH in culture media is maintained. The optimum pH for keratinase production by *Arthrobacter* sp. NFH5 was observed at pH 7.0. Similar finding was achieved from *Arthrobacter creatinolyticus* by Kate and Pethe (2014). The bacterium had a neutral pH range for keratinase production. The maximum enzyme production was also found at moderate pH by others that support the present study (Matikeviciene et al. 2011; Kim et al. 2001).

In the investigation, different inoculums volumes were tested for the production of keratinase. The production was found to increase with increasing size of inoculums and found to be optimal at 5%. Further increase in the inoculums size, greatly decreased the production might be due to rapid growth of bacteria and depletion of essential nutrients by bacteria in the early stages. Normally higher agitation rates (200–250 rpm) provided good growth of bacteria with possibly low keratinase production due to high dissolved oxygen. In contrast, substrates and bacterial cells were not well mixed at low agitation rate (100 rpm) and produced heterogeneous formation and lower dissolved oxygen meaning in low keratinase production (Pissuwan and Suntornsuk 2001).

The optimum activity of keratinase enzyme was found at pH 8.0 alongside temperature of 40 °C. Similarly, preferred pH 8.0 for activity of keratinase has been reported by others (Deivasigamani and Alagappan 2008; Inamdar et al. 2012; Saranya et al. 2015). The extracellular crude keratinases exhibited more than 54% activity at pH 7.0. Jayalakshmi et al. (2011) reported that keratinases from most bacteria have optimum pH ranging from neutral to alkaline (7.5–9.0). Meng et al. (2013) reported keratinase exhibits its optimum activity at 40 °C. Usually keratinase positive others bacteria such as *B. subtilis* exhibited optimal production at temperatures ranging from 30 to 50 °C (Mazotto et al. 2011a, b). So the properties of this enzyme increase the probability in industrial application at high temperature. *Arthrobacter* sp. NFH5 might be a good candidate for keratinase production and may be employed in feather degrading and dehairing purposes. Further studies should be carried out to purify and fully characterize the enzyme and to determine the sequence of this keratinase gene for future improvement for industrial application through genetic engineering approaches.

## Additional file

**Additional file 1: Figure S1.** Visualization of 16S rDNA PCR products by 1.2% agarose gel electrophoresis. From left side ‘M’ indicates marker DNA (1kb+), lane 1 presents genomic DNA of *Arthrobacter* sp. NFH5 and lane 2 is positive band of other bacteial strain. **Figure S2.** The 16S rDNA sequence was compared with other bacteria using online BLAST tool with deposited sequences of Genbank database. Blast result has been taken by screenshot.

## Abbreviations

KA: keratin agar
16S rRNA: sixteen subunit ribosomal RNA
DNA: deoxyribonucleic acid
MEGA6: molecular evolutionary genetic analysis version 6
PCR: polymerase chain reaction
BLAST: basic local alignment search tool
TCA: trichloroacetic acid
OD: optical density
RPM: revolutions per minute
