# Supplementary material for: Production, partial optimization and characterization of keratinase enzyme by Arthrobacter sp. NFH5 isolated from soil samples
Source: AMB Express. 2017 Sep 21;7:181. doi: 10.1186/s13568-017-0462-6 (PMC5608654; doi:10.1186/s13568-017-0462-6)

**Additional Figure Legends**

**Fig. S1.** Visualization of 16S rDNA PCR products by 1.2% agarose gel electrophoresis. From left side ‘M’ indicates marker DNA (1kb+), lane 1 presents genomic DNA of *Arthrobacter* sp. NFH5 and lane 2 is positive band of other bacteial strain.

**Fig. S2:** The 16S rDNA sequence was compared with other bacteria using online BLAST tool with deposited sequences of Genbank database. Blast result has been taken by screenshot.


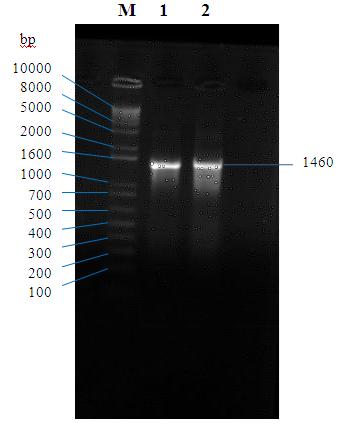


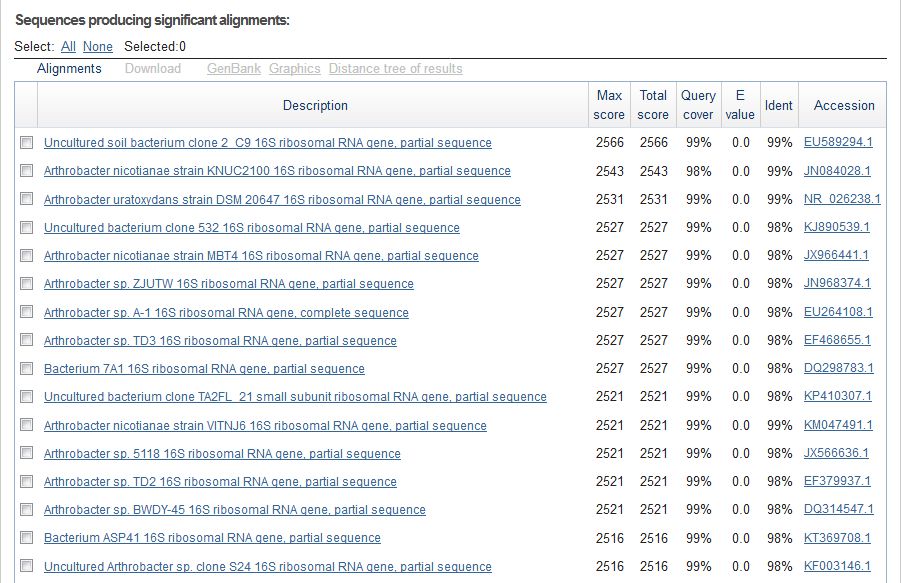

Supplement: Supplementary file 1 — Additional file 1: Figure S1. Visualization of 16S rDNA PCR products by 1.2% agarose gel electrophoresis. From left side ‘M’ indicates marker DNA (1kb+), lane 1 presents genomic DNA of Arthrobacter sp. NFH5 and lane 2 is positive band of other bacteial strain. Figure S2. The 16S rDNA sequence was compared with other bacteria using online BLAST tool with deposited sequences of Genbank database. Blast result has been taken by screenshot. [file 13568_2017_462_MOESM1_ESM.docx]
